# Strategies for Tuberculosis Prevention in Healthcare Settings: A Narrative Review

**DOI:** 10.3390/tropicalmed10110316

**Published:** 2025-11-06

**Authors:** Ahmad Faris Daradkeh, Basil Alawyia, Hassan Ballas, Nikolaos Spernovasilis, Danny Alon-Ellenbogen

**Affiliations:** 1 Department of Basic and Clinical Sciences, University of Nicosia, 2417 Nicosia, Cyprus; alawyia.bas@live.unic.ac.cy (B.A.); alon-ellenbogen.d@unic.ac.cy (D.A.-E.); 2 College of Medicine and Health Sciences, United Arab Emirates University, Al Ain 1551, United Arab Emirates; 202105602@uaeu.ac.ae; 3 Department of Infectious Diseases, German Medical Institute, 4108 Limassol, Cyprus; nikspe@hotmail.com

**Keywords:** healthcare settings, healthcare workers, infection control, prevention, Tuberculosis

## Abstract

Tuberculosis continues to represent a major occupational risk in healthcare environments, particularly for healthcare workers who have persistent contact with patients who may be infectious. Despite the high occupational burden of tuberculosis among healthcare workers, there remains a lack of focused reviews that comprehensively evaluate preventive interventions across all levels of prevention within healthcare settings. In this literature review, effective preventive interventions relevant to tuberculosis transmission have been examined. Primary preventive interventions seek to diminish exposure through protective interventions such as respirators, improvements in ventilation systems, and implementation of educational programs regarding infection control protocols. Secondary preventive interventions target early diagnosis and routine screening with efforts to detect cases and latent infections early, before they progress to active disease. Enhancements in diagnostic technology have improved both the accuracy and speed of detection, further aiding the efforts of controlling nosocomial transmission. Tertiary preventive interventions target enhancing compliance with treatment protocols, managing complications of active infection, and controlling resistant strains through individualized follow-up and interventions. Barriers like stigma and lack of resources, however, often impede such interventions’ effectiveness in many cases. This narrative literature review highlights the imperative for strengthened workplace policies, an expansion of educational programs, and continued research in new and emerging interventions like new vaccine and diagnostics technology development. All these factors aim to optimize intervention effectiveness for tuberculosis and protect the health and welfare of workers in the medical field.

## 1. Introduction

Tuberculosis (TB) remains one of the major public health issues in many parts of the world, especially in health facilities where healthcare workers (HCWs) are at increased risk of contracting the infection [1]. TB is caused by *Mycobacterium tuberculosis*, a highly communicable airborne infection spread by means of aerosolized droplets coughed into the air by active TB patients [2]. Despite the preventable nature of the disease, TB continues to infect millions across the world. According to the World Health Organization (WHO) TB report of 2024, more than 10 million people have fallen ill with TB since 2021, and the number has been rising ever since. As of 2023, 10.8 million people have been infected with TB, continuing a positive trend from 2021. This rise was mainly attributed to the COVID-19 pandemic, with the global TB incidence rate increasing by 4.6% between 2020 and 2023, from 129 in 2020 to 134 in 2023. In addition, the number of deaths attributed to TB increased during the COVID-19 pandemic from 1.34 million in 2019 to 1.40 and 1.42 million in 2020 and 2021, respectively. In 2023, the number of deaths from TB has fell to 1.25 million, a significant reduction, though still insufficient, as TB has returned to being the world’s leading cause of death from a single agent, replacing COVID-19. Overall, the global rise in TB cases globally has slowed down and started to stabilize since the COVID-19 pandemic; however, the WHO is off-track in meeting its 2025 goals. For instance, the global reduction in TB incidence from 2015 to 2023 was 8.3%, far from the WHO’s goal of 50% reduction by 2025. Moreover, the net reduction in the global number of TB-attributed deaths in the same period was 23%, far off the goal of 75% reduction set in 2015. These findings reflect the significant global burden of TB, which has been exacerbated by the aftermath of the COVID-19 pandemic, highlighting the crucial role of effective preventive strategies [3].

Moving on from global trends, in the United States, approximately 9633 TB cases were registered in the year 2023, reflecting about a 15.6% increase as compared to the cases reported across 2021. HCWs accounted for about 6.9% of the cases, highlighting the occupationally based threat existing across healthcare facilities [4]. These findings further highlight the need for effective preventive strategies in combating occupational TB.

Evidence-based interventions such as personal protective equipment incorporation, environmental controls, and education have shown effectiveness in the prevention of TB infections. For instance, the use of N95 respirators is more efficient in blocking TB droplets as compared to surgical masks [5]. In addition, environmental interventions including the use of high-efficiency particulate air filtration systems as well as ultraviolet germicidal irradiation are important for the reduction in airborne transmission of TB within health facilities. Furthermore, healthcare worker education promotes better adherence to preventive measures [6].

Routine screening as part of secondary prevention is essential for the early identification of latent tuberculosis infection (LTBI). In conditions where proper screening procedures are not followed, carrier patients can develop active TB and present a risk to HCWs as well as to patients [7]. Screening procedures like the tuberculin skin test (TST) and interferon-gamma release assay (IGRA) are mainly conducted on groups deemed to hold higher risk. Early identification with the help of molecular diagnostic tools like the Xpert MTB/RIF Ultra test facilitates implementing the required isolation and initiating treatment procedures on time, thus reducing the spread of the infection through healthcare facilities [8].

Despite these preventive measures, barriers such as stigma, fear of professional assessment, and inadequate access to services delay early identification and optimal prevention [9]. Efforts to eliminate these barriers through the enforcement of effective workplace policies, safe reporting procedures, and early availability of diagnostic services are critical to reducing the likelihood of workplace-associated tuberculosis.

Future projections suggest that without significant changes, TB prevalence will decline by only 1–2% annually—insufficient to meet End TB Strategy goals [10,11]. Even full-scale implementation of preventive therapy may only bring an 8% drop by 2030, underscoring the need for stronger, more innovative prevention policies.

At the same time, the cost burden remains a major obstacle. Around 50% of TB-affected families globally face catastrophic health costs exceeding 20% of household income [12]. In China and Ethiopia, the impact is especially severe among drug-resistant TB patients, TB-HIV coinfections, and those with limited insurance or low-paying jobs [13,14]. Indirect income loss often accounts for most of the cost, highlighting the need for preventive policies that also include financial protections and expanded access to care.

The aim of this review is to present a thorough review of TB prevention measures across healthcare settings. Key themes discussed in this review are effective strategies to reduce TB transmission across HCWs and the role played by current infection control measures to reduce workplace exposure. The review highlights the importance of strong adherence to infection control practices, early diagnosis, and focused screening activities to reduce HCWs’ exposure to TB infection.

## 2. Primary Prevention

### 2.1. Basics of Primary Prevention

Respiratory protective equipment serves as the first line of defense against infectious TB droplets with surgical masks being the most commonly used. However, they provide minimal protection due to their weak filters. On the other hand, respirators such as the N95 mask are equipped with high-efficiency particulate air (HEPA) filters, allowing for better protection. The N95 mask demonstrates a filtration efficiency of 97.4%, while surgical masks prevented only 18.4% of particles [5]. Furthermore, a study by MacIntyre et al. [6] revealed that N95 masks demonstrated greater protection against droplet-transmitted infections (RR 0.26; 95% CI 0.16–0.42, *p* < 0.001) compared to surgical masks (RR 0.65; 95% CI 0.41–1.04).

**Figure 1:**
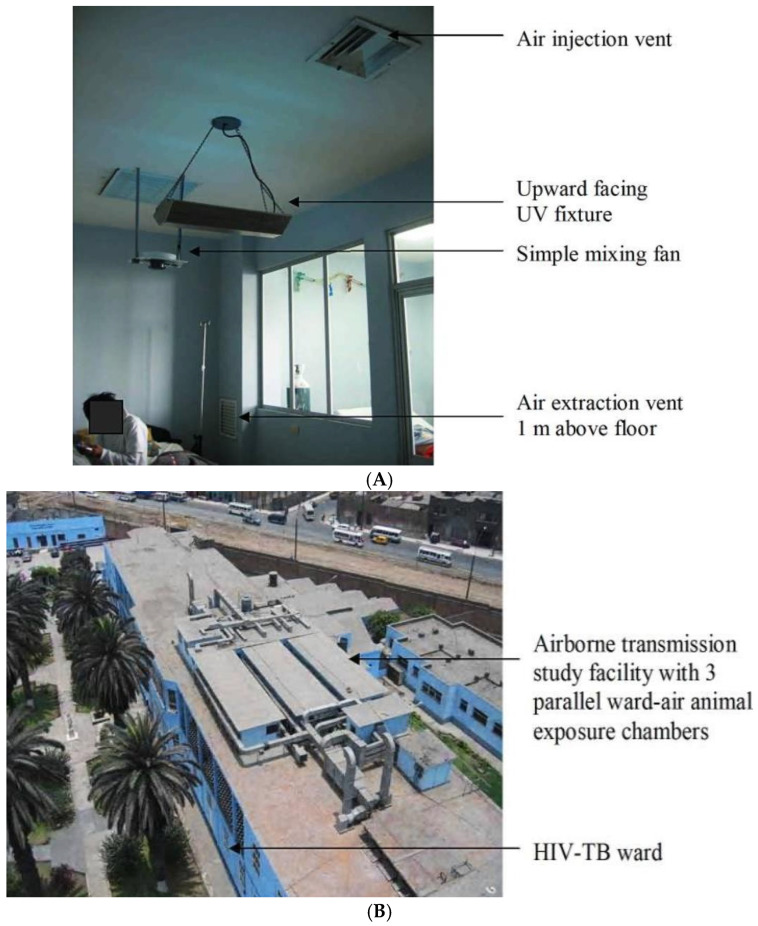
(**A**) Photograph of HIV-TB isolation room showing UV light fixture, mixing fan, and ventilation system. (**B**) Bird’s eye view of guinea pig air sampling facility on roof of HIV-TB ward.

Ventilation systems serve as a crucial, cost-effective tool in controlling TB transmission within healthcare settings. Despite their cost-effectiveness, their efficacy often depends on several climatic factors such as temperature and humidity, which could alter airflow dynamics. Where proper ventilation cannot be implemented, supplementary interventions such as techniques of air cleaning, including HEPA filtering and UVGI, can be utilized [6]. A practical study examined the effectiveness of UVGI in reducing TB transmission in healthcare facilities, illustrated in Figure 1A,B. Air from negative-pressure isolation rooms housing TB patients was directed to a roof-based facility (Figure 1A). This air passed through three separate guinea pig exposure chambers designed to simulate different conditions: untreated air in the control group, UV-treated air in the UVGI group, and ionized air in a separate chamber [15]. Specifically, Figure 1B depicts the UVGI setup within the ward, where restricted UV light fixtures were installed to prevent harmful exposure to room occupants. Fans positioned near the fixtures ensured that air in the room circulated evenly, maximizing the exposure of airborne TB bacteria to germicidal radiation. The results revealed that UVGI reduced TB transmission by 70%, significantly lowering infection rates in the guinea pigs compared to the control condition. These findings emphasize the potential of UVGI as a cost-effective solution for infection control in healthcare settings [15].

Proper hygiene protocols serve as a simple yet effective measure in infection control.

Infection control groups have emphasized proper cough etiquette and respiratory hygiene in medical settings to avert transmission of TB [16]. In addition, despite aerosol droplets being the major transmission method of TB, maintaining good hand hygiene with an effective handwashing technique and the use of soap and water is a simple yet key infection prevention practice to avoid TB [17].

### 2.2. Healthcare Worker Behaviors and Barriers Towards Infection Control

The implementation of the preventive measures mentioned above, along with other infection control policies, is highly dependent on HCW adherence, understanding, and attitudes towards these measures. As effective as they can be, they will always be hindered by poor adherence. With that in mind, understanding the barriers that prevent HCWs from adhering to infection control policies is in -itself a preventive measure. A Cochrane systemic review by Houghton et al. examined 20 studies that explored the views and experiences of nurses, doctors, and other HCWs when dealing with highly infectious respiratory diseases including TB [18]. The study presented its findings in the context of three broad domains: organizational factors, environmental factors, and individual factors. Among the organizational factors, aspects such as lack of perceived support from their management, long, ambiguous and impractical guidelines, lack of consistency of workplace policies with international policies or with what was taught before, and increased workload, particularly regarding the use of PPE, were perceived as main barriers to adherence. Regarding the environmental factors, the lack of sufficient space for isolating patients and insufficient protective equipment served as the main barriers in that domain. In most cases, these barriers were attributed to lack of funding. Lastly, failing to recognize the importance of using PPE, as well as the discomfort associated with prolonged use, hindered HCW adherence. Individually, HCWs’ fear of transmitting the infection to their families and loved ones, as well as the sense of duty towards their patients, motivated adherence to infection control policies [18].

A similar systemic review by Tan et al. [19] explored barriers to infection control in low- and middle-income countries. Some barriers, such as perceived lack of support from upper hospital management, were similar to findings in the review by Houghton et al. [18], reflecting consistent behavioral patterns despite financial differences. Nonetheless, certain barriers were more prominent in low- and middle-income countries. Issues arising from lack of funding were the most consistent finding, contributing to most of the barriers faced by HCWs. Lack of equipment, insufficient number of HCWs to run the clinic, poor infrastructure and clinic layout, and lack of isolation rooms were among the dominant barriers faced by HCWs in such settings. A study from Uganda revealed that due to poor infrastructure and improper policy implementation, TB and HIV patients were not properly separated and often seen in the same clinic, sharing the same waiting rooms [19]. Moreover, studies from Mozambique and the Dominican Republic revealed that the constant lack of equipment such as respirators leads to staff getting desensitized and accustomed to not wearing them, making implementation of infection control policies difficult. In addition to the lack of equipment, another example from South Africa revealed that some facility managers had problems understanding the written policy, and as such, they could not train their staff or clarify any misunderstandings, while other hospital managers reported a complete lack of infection control policy. This example illustrates another issue facing HCWs in low- and middle-income nations: difficulty in transitioning from policy to action. The inability to implement policies was a recurrent theme throughout the review, either due to lack of understanding of what is required, as guidelines were too vague or non-specific, or lack of authority by the clinical lead responsible for implementation [19]. Furthermore, if lack of adherence is left unrectified, it becomes embedded in the workplace culture. Throughout the review, many studies included HCWs that simply did not believe there was a need for change, as they or someone close to them had not been infected with TB. Such outlooks were observed in studies from Uganda, Mozambique, and Russia. Furthermore, poor adherence was found to be passed down from senior to junior levels. Based on a study of healthcare worker students in Uganda, 66% of students feared academic consequences if they spoke up. All these factors come into play to create a work culture that is resistant to change. A study from South Africa reported a sense of powerlessness to change among HCWs, with many believing things are broken beyond repair. All these factors foster a work culture that is resistant to change and serves as a significant barrier to infection control [19]. On the other hand, both reviews by Houghton et al. [18] and Tan et al. [19]. illustrated the negative effects of lack of funding on infection control, which was worse in low- and middle-income countries. The review by Tan et al. [19] highlighted the effects of a poor economy on the behaviors of HCWs, and how over time it can create a work culture that acts as a barrier to infection control and prevention.

Furthermore, cultural and linguistic differences among patients and staff can create communication barriers that can contribute to poor infection control. This has been examined by a phenomenological study of the experiences of South African nurses in TB prevention and control by Sissolak, D. et al. [20]. The study revealed that language barriers can delay the diagnosis of TB and can make the understanding of the diagnosis and treatment difficult. This issue is particularly relevant to high-burden countries such as South Africa and India, where multiple languages exist, or to countries with a high number of immigrants. Moreover, cultural competency plays a key role in understanding patients’ motives and aiding them in understanding their condition and treatment. The majority of the nurses in the study were concerned that most patients went to traditional healers first to aid them. None of the nurses in the study stated they would ever refer a patient to such healers. On the other hand, some nurses were prepared to work with these healers if the patient requested it, as they believed these healers often had greater medical authority over the patient compared to them. This approach illustrates a degree of cultural competency that allows for cultural barriers to be overcome. Furthermore, similar language and cultural barriers may exist among HCWs, further contributing to poor infection control. Consequently, understanding the social structure of the city, town, or area can help overcome many barriers to infection control among patients and staff [20].

Despite TB being a curable disease, the stigma towards it has not changed. In most cases, stigma toward HCWs infected with TB originates from their colleagues within the healthcare workforce, most of which is attributed to the perceived risk of transmission. HCWs diagnosed with TB are often avoided or excluded, despite presenting a very low infection risk. Moreover, TB has been associated with malnutrition, poverty, and HIV, which may prompt speculations about the HCWs immune status and general lifestyle, further fueling the stigma. On the other hand, patients may direct stigma towards themselves with feelings of shame and isolation, which are often associated with the need to use a mask to reduce transmission. Such stigma, internal or external, may cause infected HCWs to become reluctant to engage in care [21].

### 2.3. Suggested Solutions

In 2017, the Umoya Omuhle project was launched by the London School of Hygiene and Tropical Medicine with the aim of developing effective interventions that can help in overcoming the barriers mentioned in the previous studies [22]. The project adopted a multidisciplinary approach focusing on areas such as policy analysis, epidemiological studies, spatial mapping and infrastructure assessment, and ethnographic research. Parameters such as number of contacts made per visit, probability of a patient being infectious, and contact duration were considered. Additionally, the movements of people within clinics and the organization of queues or outpatient waiting areas were also accounted for. Using a mathematical model based on these data, various interventions were developed that could potentially improve infection control in these settings [22]. Some of the top-ranking interventions in the Umoya Omuhle Project were implemented in a study by McCreesh et al. [23]. The study aimed at assessing the efficacy and practicality of implementing these interventions in public health clinics. In order to remain consistent with the Umoya Omuhle project, clinics in KwaZulu-Natal and Western Cape provinces in South Africa were selected. Interventions such as opening windows and doors reduced the transmission rate by 55% (IQR 25–72%), clinic retrofits by 45% (IQR 16–64%), installing UVGI by 77% (IQR 64–85%), patients wearing surgical masks by 47% (IQR 42–50%), and a queue management system plus outdoor waiting areas by 83% (IQR 76–88%). Interestingly, queue management systems, such as appointments and outdoor waiting areas, have shown the highest effectiveness in reducing transmission, particularly among clinic staff other than HCWs, such as security officers and secretaries, reflecting the need to broaden the perspective beyond traditional infection control measures. Furthermore, the practicality, ease, and cost of these interventions have to be considered when implementing them, which will vary among different settings. Factors such as sufficient space, appropriateness of the outdoor climate enabling outdoor waiting areas, the availability of appropriate staff to manage implemented interventions, and sufficient financial resources must be considered. Applying the appropriate interventions in the context of these factors is key to ensuring their effectiveness is not lost [23].

Although adequate protective equipment, clinic infrastructure, and staffing are fundamental to infection control, they are insufficient for addressing the critical role of healthcare worker behavior and workplace culture. While the former can be solved by increasing funding, the latter will not benefit as much [19]. Understanding the reasoning behind these behaviors can aid in developing solutions to them. In both reviews [18,19], some behavioral patterns resonated with several behavioral theories such as the health belief model and social-cognitive theory. According to the health belief model, understanding the risks in combination with low barriers to protection and high perceived benefits from action drives people to alter their behavior [24]. This can be promoted via reducing barriers to protection similar to what was achieved in the Umoya Omuhle project, as well as ensuring that HCWs receive adequate training. However, it is important to note that despite training being a recurring theme in both reviews [18,19], a review by Ward D. J. [25] revealed that while additional training does improve knowledge, it does not always effectively change practice. This has been attributed to a gap between knowledge and application, a widely observed phenomena in healthcare and a recurring theme in the review by Tan et al. [19]. On that basis, ensuring that the appropriate measures are applied is key to benefiting from additional training; otherwise, it loses its effectiveness [25]. Understanding why a knowledge-action gap exists in healthcare is difficult, as there are many factors to consider. The social cognitive theory attempts to solve this by suggesting that, while knowledge of health risks is important for change, personal beliefs about oneself and their surroundings are key for change. Personal efficacy to change, expected social approval or disapproval to the change, and the perception of certain barriers to change are all personal beliefs that can affect behavior. While these theories provide some explanation as to why people behave in a certain way, they are often generalized and may not always apply to specific contexts as they do not always consider cultural, social, and economic factors, as well as the effect of the unconscious habits. Nonetheless, using these theories, among others, in combination can aid in the understanding of health behavior and assist in the design of behavioral change interventions [24].

### 2.4. Role of Vaccines

In 1921, the Bacillus Calmette–Guerin (BCG) vaccine was approved for clinical use against TB. It remains the only vaccine approved for this indication. The WHO added the BCG vaccine to its expanded program on immunizations in 1974; however, only a few countries with a high TB burden have included it in their universal vaccine strategies, while in other countries, it is recommended for high-risk groups. The lack of universal use of the vaccine is mainly attributed to its debated effectiveness in protecting against TB [26]. A meta-analysis by Martinez et al. [27] assessing the efficacy of the BCG vaccine revealed that its overall effectiveness was 18% (aOR 0.82, 95% CI 0.74–0.91). When stratified by age, the BCG vaccine was most effective in children 5 years or younger (aOR 0.63, 95% CI 0.49–0.81). These results reflect the need for new vaccination strategies, as the BCG vaccine fails to protect older populations [27].

The rapid development of bioinformatics technology in 2010, combined with the rise of COVID-19 peptide-based vaccines in 2020, shifted much attention to peptide vaccines for TB. Several factors, such as the ability to induce a strong immune response via immunodominant epitopes, good stability, low cost, easy of storage and transport, and decreased side effects, make peptide vaccines promising vaccine candidates [28]. In 2019, the results of a Phase IIb clinical trial of the M72/AS01_e_ revealing an overall effectiveness of 54% (95% CI 2.1–74.2) were published [28]. Many other peptide vaccines are currently in development or undergoing clinical trials. As the results of these trials are published, more insights can be gained regarding the effectiveness, safety, and generalizability of peptide-based vaccines within different populations [28].

In addition to peptide vaccines, several other novel TB vaccines have entered the clinical trial pipeline and are now under evaluation. Two live vaccines, the MTBVAC, a genetically attenuated Mtb, and VPM1002, a genetically enhanced BCG vaccine, are under clinical evaluation (NCT04975178) and (NCT04351685), respectively [29]. Furthermore, the BNT164a1/BNT164b1 is a major mRNA TB vaccine currently undergoing Phase 1 clinical trials (NCT05547464/NCT05537038). It remains to be seen whether these new technologies will be integrated into primary TB prevention [29].

Figure 1A: In a negative-pressure isolation room equipped with upper-room UVGI, airborne infection control is achieved without exposing patients or staff to hazardous levels of UV. Each room contains a luminaire fitted with two 9-watt UV bulbs housed in a baffled casing that confines high-intensity UV radiation to the upper airspace, safely away from occupants. Adjacent to this fixture is a mixing fan capable of moving 382 m^3^ of air per hour, which enhances vertical circulation and increases the likelihood that airborne TB bacilli are drawn into the irradiated zone. Ventilation is further optimized by a ceiling-mounted supply vent and an extraction vent positioned at approximately one meter above the bed, with both vents placed based on computational fluid dynamics modelling to ensure proper airflow patterns. This arrangement disinfects exhaled air in the upper part of the room while maintaining negative pressure and directing air safely toward the rooftop exposure chambers [15].

Figure 1B: To investigate airborne tuberculosis transmission in a realistic clinical setting, a dedicated facility was constructed on the roof above the HIV-TB ward. Four negative-pressure isolation rooms on the ground floor housed TB-HIV co-infected patients and supplied ward air to the rooftop via ducted exhaust. This air was then directed into one of three parallel exposure chambers (each approximately 70 m^3^ and maintained under negative pressure) containing up to 150 guinea pigs, animals chosen for their high susceptibility to Mycobacterium tuberculosis. After passing over the animals, the air was sterilized and vented through chimneys. By delivering untreated ward air directly to the enclosures and avoiding deliberate passage through UV fields, the system provided a true-to-life model for evaluating interventions such as upper-room UV irradiation and negative air ionization [15].

## 3. Secondary Prevention

### 3.1. Routine Screening

**Figure 2:**
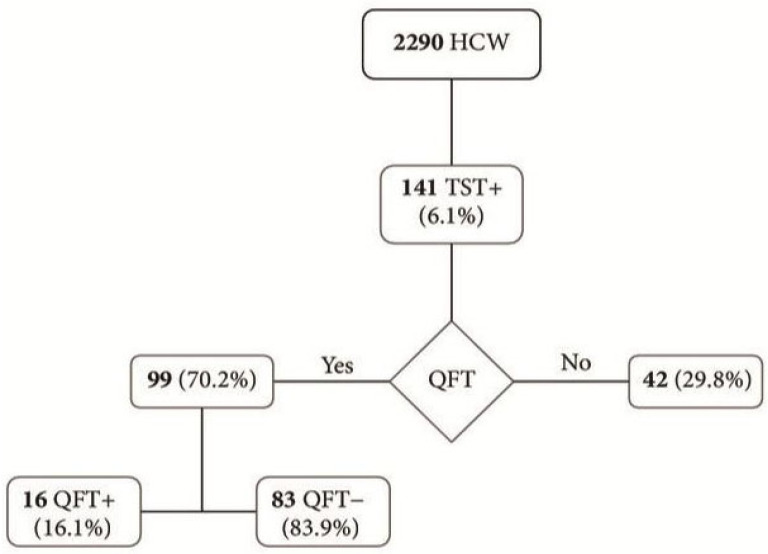
Flowchart showing the study population, TST, and IGRA results [27].

To illustrate practical approaches to TB screening among HCWs, a notable study conducted in Rome provides valuable insight into the implementation of a two-step diagnostic protocol. This study analyzed TB screening in HCWs at a local teaching hospital over a period of three years. The key purpose of this research was to evaluate the effectiveness of a two-step screening measure in diagnosing latent TB infections [30]. The research produced several key findings (shown in Figure 2). Firstly, in the screening protocol, 2290 HCWs underwent initial testing using the tuberculin skin test (TST), of whom 141 (6.1%) tested positive. Those with a positive test result were then further analyzed using QuantiFERON-TB Gold (QFT), an interferon gamma release assay (IGRA). Among these, 16 cases (16.1%) were confirmed positive, while the majority (83 individuals) tested negative. The flowchart highlights the screening progression and diagnostic pathways, showcasing how test results require further action [30].

Subsequently, the study emphasized the value of IGRA in screening, depicting its use with increased specificity over TST. With increased specificity, rates of false positives diminished, particularly in populations previously vaccinated by the BCG vaccination. Finally, the study showed that in low-prevalence settings, the use of TST as a first-step screening method followed by an IGRA as confirmatory testing proved efficient and effective [30].

While universal TB screening among HCWs is globally recommended, its practice among health institutions remains far from consistent. Research conducted across both high- and low-income countries reports irregular testing schedules, minimal occupational-health coverage, and deficient monitoring frameworks hindering follow-up among positive cases [31,32,33]. In low-resource settings, shortages of reagents and trained staff limit the feasibility of IGRA-based screening, whereas in high-income nations, declining compliance among long-term employees is common [19,31].

Recent economic evaluations have demonstrated that the cost-effectiveness of IGRA screening is highly dependent on local TB incidence, becoming economically favorable only in higher-risk populations, and that routine annual IGRA testing is not cost-effective in low-prevalence settings [34,35]. Moreover, a cost-effectiveness study in Italy showed that annual IGRA screening incurred substantial expenses (approximately EUR €39,000 per seroconversion), highlighting its limited value when used universally and suggesting that in resource-limited settings, IGRA testing should be reserved for high-risk or highly suspected cases [36].

In settings where IGRA screening is deemed cost-effective, sustainable implementation requires institutional commitment, continuous funding for consumables, digitalized tracking systems for test results, and clear alignment with national TB-prevention policies [32,33]. These implementation priorities align with recommendations from both the WHO 2020 Preventive Treatment Module and the CDC 2024 TB screening guidelines, which emphasize risk-based, context-appropriate screening and the integration of rapid diagnostics into occupational-health programs [32,33].

### 3.2. Early Diagnosis

Early diagnosis is critical for preventing TB, particularly in medical settings. In such settings, delayed diagnosis can lead to dire consequences, e.g., hospital-acquired tuberculosis among vulnerable patient populations (e.g., immunocompromised individuals: patients undergoing chemotherapy, HIV/AIDS, or candidates for organ transplantation) [37]. Ultimately, delays in diagnosing infectious cases increase the risk of transmission, exacerbating the danger for both HCWs and patients in general [38].

Regular testing using TST or IGRA remains an essential tool for detecting latent TB infection before progression to active disease, particularly among HCWs in high-risk departments such as pulmonology, intensive care, and TB clinics [6]. Early detection enables prompt intervention and has been shown to significantly reduce workplace transmission rates.

Moreover, identifying HCWs who are at higher risk of contracting TB further aids in early diagnosis. A multivariate parametric exponential model evaluated age, job role, work setting, and sex in relation to TST conversion among HCWs. In this analysis, sex did not influence the hazard of conversion; the adjusted hazard ratio for male versus female workers was 0.99 (95% CI 0.8 to 1.2). Workplace, however, was a significant determinant of risk. Using medicine services as the reference category, workers assigned to surgery (HR 1.59), infectious disease wards (HR 1.94), and laboratories (HR 2.84) faced significantly higher hazards, whereas those in outpatient clinics showed no difference from the reference group. Estimates for administrative staff were unstable because of the small number of conversions. Occupational category was also associated with risk: relative to nursing staff, physicians had about a 16% lower hazard of conversion, and social workers had about a 68% reduction. Finally, increasing age conferred a slight elevation in risk, approximately a 3% increase in hazard for each additional year of age [39].

However, despite its importance, several barriers hinder widespread adoption. Stigma, fear of isolation, confidentiality concerns, and scheduling disruptions may lead HCWs to avoid screening or conceal symptoms [31]. These challenges underscore the need for confidential, institutionally supported screening policies and targeted educational initiatives that normalize testing and enhance TB awareness among healthcare personnel [31,40].

Recent analyses further emphasize the importance of integrating cost-effective diagnostic strategies into early detection programs. Comparative economic evaluations of molecular diagnostic tools, such as Xpert Ultra, TB-LAMP, and TB-LAM, found that the optimal choice depends strongly on baseline TB prevalence [41]. Similarly, d’Elbée et al. (2024) demonstrated that decentralizing molecular testing to peripheral facilities can reduce diagnostic delays and yield favorable cost-effectiveness ratios, provided that sufficient infrastructure and training are in place [42]. These findings highlight the need for context-specific diagnostic planning rather than uniform implementation across all institutions. Global guidelines reinforce these priorities.

The CDC 2023 update supports baseline TB screening for all newly employed HCWs using a combination of risk assessment, computer-aided chest radiography, and molecular diagnostic tools to ensure timely detection in high-burden settings [32]. The WHO Module 1 (2020) on tuberculosis preventive treatment similarly advocates for risk-based screening strategies tailored to both high- and low-incidence contexts, particularly among HCWs with repeated occupational exposure [33]. Collectively, these approaches mark a shift from universal annual screening to targeted, evidence-informed early diagnosis strategies that balance effectiveness, feasibility, and cost-efficiency in real-world healthcare environments.

### 3.3. Role of Rapid Diagnostics

A recent study evaluating the Xpert MTB/RIF Ultra (Xpert Ultra, an enhanced molecular diagnostic tool) highlighted its value in improving TB detection in clinical settings. Compared to the standard Xpert MTB/RIF assay, Xpert Ultra demonstrated superior sensitivity, particularly in smear-negative and HIV-positive patients, while maintaining high specificity [8]. This improvement in diagnostic performance supports faster and more accurate identification of TB, which is crucial in healthcare environments where HCWs and vulnerable patients face elevated exposure risks. By enabling earlier diagnosis, Xpert Ultra strengthens infection control measures and contributes to reducing TB transmission within medical facilities [43].

In parallel, recent innovations in artificial intelligence (AI) have further enhanced TB screening. Deep learning models applied to chest X-rays have achieved diagnostic accuracies exceeding 92% sensitivity and specificity, surpassing human radiologists in high-burden settings and meeting WHO triage standards [44]. Their deployment may reduce dependence on confirmatory molecular tests, such as GeneXpert, by as much as 50%. Additionally, whole-genome sequencing (WGS) is reshaping drug-resistant TB management [45]. A global WHO analysis of 38,000 isolates catalogued over 13,000 mutations, including 1149 associated with resistance to 13 anti-TB drugs. This comprehensive mutation database now supports resistance prediction and can be integrated into routine diagnostic workflows to guide personalized treatment [46].

Although such technological breakthroughs are key milestones, their effectiveness in real-world applications lies in their practical feasibility and economic effectiveness. Introduction of such equipment is dependent on ongoing investment in infrastructure at a laboratory level, maintenance processes, and staff training—all elements that are frequently deficient in low-income settings [19,32]. Clinical trials have identified that the integration of low-capacity triage methods with molecular testing maximizes both diagnostic yield and economic effectiveness, making interventions scalable that take place within high-burden settings [47,48]. Development of systematic evaluability standards that consider cost, infrastructure readiness, and functional feasibility can support health facilities in their technology priorities with long-term potential [31,32]. Aligning these adoption processes with the WHO End TB Strategy (2023) and CDC TB screening guidelines (2024) ensures rapid diagnostic preventive measures, thereby enhancing early detection and promoting comprehensive TB prevention across HCWs [32,33].

Figure 2: In this TB surveillance cohort, HCWs who were TST-positive (141) underwent a two-step evaluation. Among those with a positive TST, 99 (about 70%) had follow-on QuantiFERON-TB testing (introduced in May 2014) and 16 (16.1%) were confirmed positive. IGRA positivity was 12.8% in 2014 and 18.3% in 2015, with a median interferon-γ level of 5.5 IU/mL. Neither TST nor IGRA positivity varied by work area; however, both were strongly linked to documented occupational exposure. BCG vaccination increased the odds of TST positivity but was not associated with IGRA positivity and appeared protective in multivariate analysis. TST positivity increased significantly in 2015 compared with 2013–2014, independent of the type of tuberculin used, whereas IGRA positivity rose slightly but not significantly. Concurrently, reported TB cases in the hospital declined, indicating that the rise in TST positivity likely reflected true latent infection rather than an increase in active disease [30].

## 4. Tertiary Prevention

### 4.1. Ensuring Adherence to Treatment

Adherence to TB treatment is a critical determinant of therapeutic success and the prevention of drug resistance. Interruptions or deviations from the prescribed regimen can lead to treatment failure, prolonged infectiousness, and the development of resistant TB strains. Directly Observed Therapy (DOT), wherein HCWs supervise patients during medication intake, remains a widely implemented strategy to ensure adherence [39]. Evidence supports its effectiveness in reducing nonadherence, a major contributor to unsuccessful outcomes. In clinical and occupational settings, especially among HCWs, adherence strategies like DOT are essential to maintaining the integrity of treatment protocols and achieving disease control [49]. A modern and emerging alternative to DOT for TB treatment is Video-Observed Therapy (VOT). While both methods aim to encourage patients to adhere to their treatment, VOT offers an advantage due to its portable approach. VOT uses digital technology, such as video recording, which reduces the need for hospital visits and minimizes HCWs’ work time while still maintaining adherence. Moreover, VOT offers patients greater flexibility and convenience, which increases satisfaction without compromising care [50].

For HCWs, self-administered therapy (SAT) is a viable alternative when DOT is not feasible. It involves strong supportive systems, including follow-up appointments, reminders for medications, and other interventions [51]. SAT also enables HCWs to deliver therapy effectively, even when challenged with hectic workloads, while at the same time maintaining compliance with protocols in practice. In 2020, a study emphasized the disadvantages of using a facility’s DOT during the COVID-19 pandemic and proposed alternatives in terms of community DOT, video DOT, and SAT. Not only did these alternatives improve access and compliance, but they also reduced the transmission of TB. According to the study, SAT is a feasible alternative for HCWs with hectic work schedules, allowing for therapy integration into their routine, thus contributing to a possible reduction in transmission of TB in healthcare environments [50]. 

Ensuring consistent adherence to treatment among HCWs is therefore not only essential for individual recovery but also a cornerstone of institutional TB prevention. Strengthening adherence strategies such as DOT, VOT, and SAT directly supports infection control efforts, protecting both healthcare workers and the patients they serve.

### 4.2. Drug-Resistant TB

Extensively Drug-Resistant Tuberculosis (XDR-TB) is a severe form of TB characterized by resistance to rifampicin, any fluoroquinolone, and at least one Group A drug such as bedaquiline or linezolid. Although it constitutes a relatively small proportion of total TB cases, XDR-TB is associated with significantly higher treatment complexity, prolonged duration, and reduced therapeutic success [52] In 2020, the WHO estimated approximately 500,000 new cases of rifampicin-resistant TB globally, yet only 157,903 were detected and reported, of which 25,681 were classified as pre-XDR or XDR-TB. This represents a substantial gap in case detection and management [53]. The COVID-19 pandemic further exacerbated this deficit, contributing to a 22% decrease in drug-resistant TB case notifications and a 15% reduction in treatment enrollment from 2019 to 2020 [52].

The emergence and spread of XDR-TB are driven by several factors, including suboptimal treatment adherence, incomplete or inadequate therapeutic regimens, limited access to second-line drugs, and insufficient implementation of drug susceptibility testing, particularly for newer agents. These conditions have facilitated the development and transmission of highly resistant TB strains [52].

Molecular evidence shows that XDR-TB isolates maintain their potential for transmission even when resistant, resulting in continued epidemics in parts of South Africa, India, and Iran [52] Treatment outcomes for XDR-TB remain suboptimal despite the use of newer drugs like bedaquiline and delamanid. According to recent data, treatment success in delamanid-containing regimens averaged 80.9% in observational studies but dropped to 72.8% when combined with bedaquiline, reflecting the complexity of cases requiring both drugs [54]. In a global cohort of 883 patients, including over 30% with XDR-TB, success rates for bedaquiline-based regimens were 74.2%, with a 6.5% mortality rate. These discussions highlight the limited efficacy of current regimens in real-world, drug-resistant TB settings [54].

As highlighted in a 2025 review by Matteelli et al. [55], the prevention of MDR and rifampicin-resistant TB through targeted tuberculosis preventive therapy (TPT), particularly the recently WHO-endorsed 6-month levofloxacin regimen, has emerged as a vital and cost-effective strategy for halting transmission among high-risk contacts. Given the rising complexity of treating drug-resistant strains and the limitations of current regimens for pre-XDR and XDR-TB, the authors conclude that ultimately, effective control of drug-resistant TB depends on both robust treatment strategies and strong preventive measures within healthcare settings. Strengthening adherence, early detection, and infection control practices among HCWs is crucial to limiting the spread of resistant strains and protecting HCWs from occupational transmission [55].

## 5. Cost-Effectiveness and Real-World Applicability

The success of TB prevention in health facilities relies as much on clinical effectiveness as on the economic and practical feasibility of interventions. At the primary level, environmental control measures such as improved ventilation, UVGI systems, and administrative measures showed cost-effectiveness in reducing nosocomial transmission. In low- and middle-income countries, where infrastructure is often limited, even modest ventilation upgrades can substantially decrease transmission at relatively low cost, while in high-income settings, optimizing existing systems and ensuring maintenance yields greater long-term savings [41,42]. In the context of secondary prevention, cost-effectiveness depends largely on the choice and frequency of diagnostic tools. Studies show that while IGRA offers superior sensitivity compared to TST, routine annual testing is not economically justified in low-incidence, high-income countries [34]. In contrast, risk-based or targeted screening models are more feasible and efficient in resource-limited settings [51]. Integrating affordable triage methods, such as symptom-based screening or AI-assisted chest radiography with molecular diagnostics, improves early detection while maintaining financial feasibility in both contexts [47,48]. This flexible approach enables health systems to optimize diagnostic yield according to their resource capacity. At the tertiary level, innovative adherence strategies such as VOT reduce operational costs and maintain treatment compliance, proving feasible in both high-resource and low-resource environments [42,50]. Ultimately, integrating cost-effective, context-sensitive interventions ensures that TB prevention remains both achievable and sustainable. Aligning these approaches with the WHO End TB Strategy (2023) and CDC TB screening guidelines (2024) strengthens institutional capacity, protects HCWs, and supports long-term progress toward TB elimination [32,33].

## 6. Discussion and Conclusions

TB remains a significant occupational hazard for HCWs, especially in environments characterized by insufficient infection control measures and constrained resources.

Primary measures such as respiratory protective equipment, environmental controls, and workplace hygiene protocols form the foundational layer of defense. However, the effectiveness of these interventions is ultimately dependent on the behavior, knowledge, and adherence of HCWs. System and behavioral disincentives to proper infection control include, amongst others, resource insufficiency, vague policies, and enduring stigma.

Secondary prevention measures, such as routine screening, early diagnosis, and rapid molecular testing, are essential for diminishing TB transmission within healthcare environments. Enhanced diagnostic tools like the Xpert MTB/RIF Ultra and AI-based radiographic screening offer significant improvements in early detection and targeted intervention.

Tertiary interventions like adherence supervision (including DOT and VOT), rehabilitation interventions, and nutrition supplementation also play valuable roles in decreasing disease complications, managing drug-resistant disease, and facilitating safe and effective clinical work resumption among HCWs. Moreover, emerging vaccines, particularly peptide- and mRNA-based formulations, represent a promising advancement in protection against TB, especially in adult populations where current BCG strategies are insufficient.

Prevention of TB involves an integrated and multi-faceted approach at multiple levels. The prevention of TB among HCWs is both an ethical responsibility and a public health imperative, as the well-being of HCWs serves as the cornerstone for maintaining and sustaining effective healthcare delivery systems. The integration of innovative tools, behavioral science, and targeted, evidence-based systemic approaches at the highest levels should serve as the foundation for future interventions aimed at TB prevention and workplace safety among HCWs on a global scale.

In light of the evidence provided, it is recommended that healthcare institutions adopt a more integrated prevention model that combines environmental, administrative, and behavioural measures to reduce occupational TB transmission. Promoting adherence among HCWs through continuous education and a supportive workplace remains crucial to the success of these strategies. The use of digital adherence technologies and structured monitoring systems could further enhance screening consistency and treatment completion rates. This review aligns closely with the current WHO Consolidated Guidelines on Tuberculosis: Module 1 Prevention (2024) and the CDC tuberculosis screening, testing, and treatment of U.S. health care personnel guidelines (2019), both of which emphasize risk-based screening, rapid diagnostics, and the protection of HCWs. However, notable gaps persist between these global recommendations and their application in clinical practice. Limited diagnostic infrastructure in low-resource settings, inconsistent adherence to infection control protocols, and the absence of behavioural and institutional mechanisms to ensure compliance remain key challenges. These gaps underline the importance of adapting international guidelines to local health system capacities, ensuring that preventive strategies remain both feasible and sustainable within real-world healthcare environments.

## Data Availability

No new data was provided as this is a literature review.

## Abbreviations

The following abbreviations are used in this manuscript:

TB	tuberculosis
HCW	healthcare workers
LTBI	latent tuberculosis infection
TST	tuberculin skin test
IGRA	interferon-gamma release assay
HEPA	high-efficiency particulate air
UVGI	Ultra-Violet Germicidal Irradiation
PPE	personal protective equipment
BCG	Baccillus Calmette-Guerin
QFT	QuantiFERON-TB Gold
CAD	computer-aided detection
AI	artificial intelligence
WGS	whole-genome sequencing
DOT	Directly Observed Therapy
VOT	Video-Observed Therapy
SAT	self-administered therapy
XDR-TB	Extensively Drug-Resistant Tuberculosis
TPT	targeted tuberculosis preventive therapy
